# A double-suture cerclage reduction technique with Nice knots for comminuted patella fractures (AO/OTA 34-C3)

**DOI:** 10.1186/s13018-023-03574-2

**Published:** 2023-02-16

**Authors:** Yanchun Gao, Xiaojun Han, Bin Zhou, Shichang Zhao, Xingang Yu

**Affiliations:** 1 Department of Orthopedic Surgery, Shanghai Sixth People’s Hospital, Shanghai Jiao Tong University, Shanghai, 200233 China; 2 Department of Orthopedics, Qingpu Branch of Zhongshan Hospital Affiliated to Fudan University, Shanghai, 201700 China; 3 Department of Orthopedic Surgery, People’s Hospital of Bazhou, Xinjiang, Korla, 841000 China

**Keywords:** Comminuted patellar fracture, Nice knots, ROM, Böstman score

## Abstract

**Background:**

Comminuted patella fractures place high demands on surgeons’ surgical skills. We used a double-suture cerclage reduction with Nice knots as an intra-operative reduction technique to displaced comminuted patella fractures.

**Methods:**

Patients were divided into two groups by whether or not an intra-operative suture cerclage reduction technique was used. Fragments count, surgical time, quality of the reduction, and fracture healing time were recorded. The postoperative function was assessed by Böstman score and range of motion.

**Results:**

With the inclusion and exclusion criteria, 48 patients we included in the cohort between Sept. 2016 and Oct. 2021. The double-suture cerclage reduction technique with a Nice knot achieved a satisfactory reduction. When the number of fragments was over 5, this technique showed significant advantages in saving surgery time.

**Conclusions:**

In this study, the double-suture cerclage reduction technique combined with the Nice knot shows significant advantages for displaced highly comminuted patella fractures. This technique simplifies the operation and saves surgical time, which is helpful for clinical practice.

## Introduction

Patella fractures, accounting for about 3.5% of lower extremities fractures, mainly result from high-energy trauma and can result in stiffness, patellofemoral arthritis, and disabling sequelae if not treated appropriately [1, 2]. Comminuted patella fractures comprise about 55% of operatively managed patellar fractures [3–5]. The vast majority of patellar fractures are intra-articular, which places a high demand on the fracture repositioning of the patella. It remains a challenge to orthopedic surgeons.

The surgical treatment of patellar fractures aims to accurately reposition the fragments and restore the quadriceps extensor mechanism and knee function [6]. The comminution was thought to hinder the accurate reduction of the articular surface. Poor fracture reduction can often lead to many post-operative complications, including delayed union or nonunion, limited knee function, and post-traumatic osteoarthritis [4, 7]. Prolonged operative time due to comminuted articular surfaces was also associated with post-operative infection and wound complications [8]. Although numerous studies have reported on the internal fixation methods of comminuted patella fractures with favorable outcomes, the repositioning technique for high-comminuted patella fractures is lacking in detail.

The Nice knot is a sliding and self-locking double-stranded knot, which is a valuable tool for reducing and fixing bone fragments [9]. It demonstrates suitable clinical biomechanical characteristics [10]. Although the Nice knot is widely used in several fractures, its application in intra-operative fracture reduction is rarely mentioned in comminuted patellar fractures [11–13].

In the present study, we used a double-suture cerclage reduction with Nice knots as an intra-operative reduction technique to displaced comminuted patella fractures. This study aimed to assess whether this low-cost, easily performed technique could lead to shorter operative time, fewer intra and post-operative complications, and better post-operative clinical outcomes.

## Patient and methods

### Ethical approval

This is a retrospective cohort study. The ethics committee at Shanghai Sixth People’s Hospital approved the study. Informed consent was obtained from all participants included in the study. In addition, this study was conducted per the Code of Ethics of the World Medical Association (Declaration of Helsinki) for human procedures.

### Patients

We retrospectively assessed 77 patients who underwent open reduction internal fixation surgery for comminuted patellar fractures at our institution from Sep.2016 to Oct. 2021.

The inclusion criteria were as follows: (1) patellar fracture of the AO/OTA classification of 34-C3 (2) Age ≥ 18 years and≤ 65 years. (3) Fresh patella fracture.

The exclusion criteria were as follows: (1) undisplaced comminuted patella fractures, (2) primary knee disease, deformity, or functional limitation, (3) severe multiple injuries to the brain, chest, or abdomen; (4) patients with serious medical diseases or complications, such as diabetes, hypertension, and heart disease; (5) open patella fractures.

AO/OTA patellar fracture classifications were independently determined by two senior orthopedic surgeons based on imaging results on CT scanning results [14]. Patients were divided into two groups by whether or not an intra-operative suture cerclage reduction technique was used. Three experienced orthopedic trauma surgeons performed surgery on patients according to the surgeons’ schedule.

### Surgical techniques and post-operative management

The patients were routinely given limb nerve block and inhalation anesthesia in the supine position. With the application of a tourniquet, patients in group A underwent the traditional reduction. After the skin incision and exposure of the whole patella, the periosteum and patellar tendon enclosing the patellar fracture fragments were opened. After thorough debridement of the soft tissue and blood clots in the fracture gap, we repositioned each fragments using point repositioning forceps, followed by temporary fixation with K-wires.

**Fig. 1:**
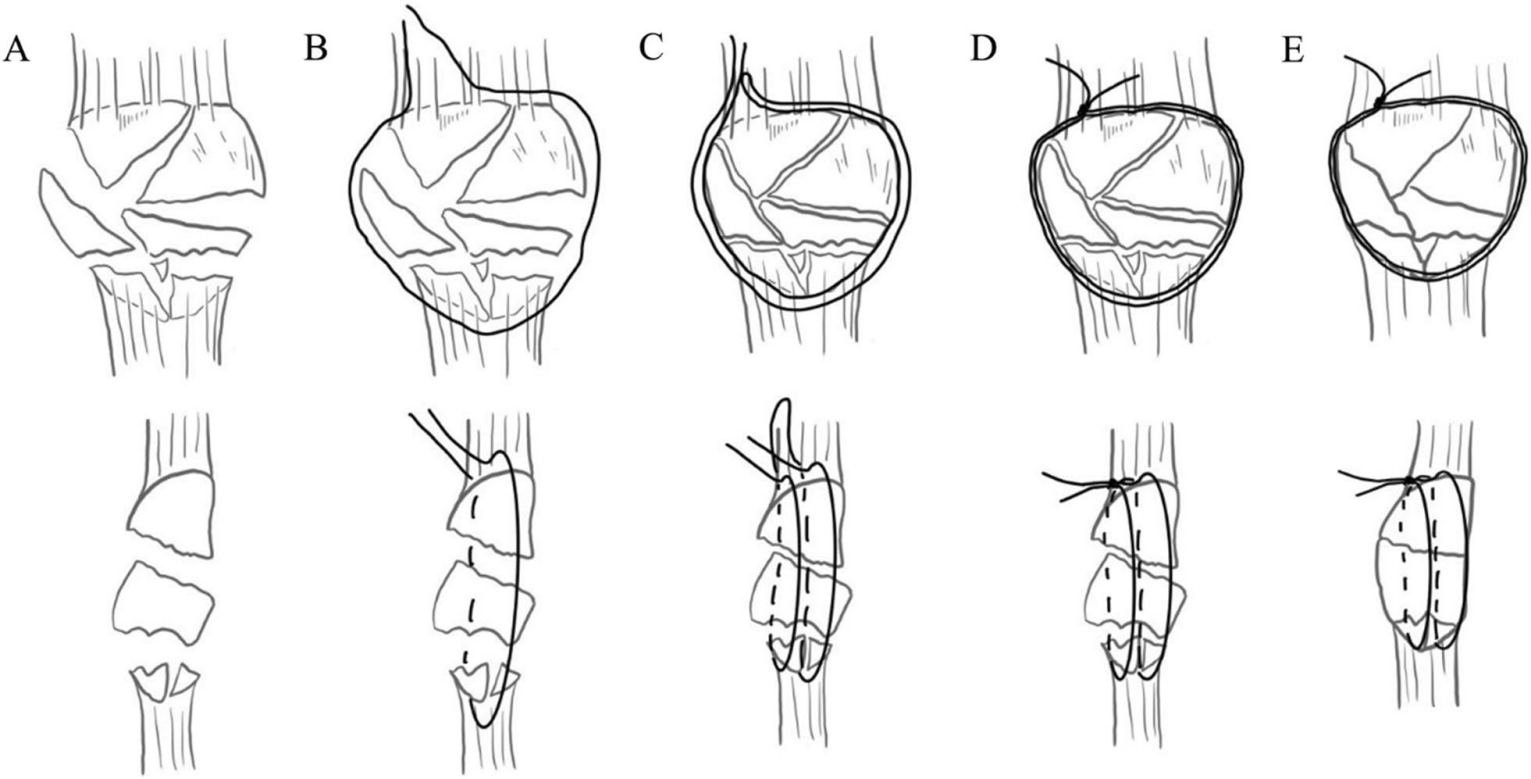
**A** Highly comminuted patellar fracture **B** Counterclockwise cerclage at two-third of the depth of the patella **C** clockwise cerclage at one-third of the depth of the patella **D** use of Nice knot and repositioning of the fragments **E** tightening of the cerclage sutures

In group B, the patients underwent open reduction and internal fixation of patella fractures with the double-suture cerclage reduction technique. The integrity of the periosteum and the patellar tendon persevered, and the medial or lateral retinacula of the patella were open. The joint cavity was thoroughly flushed with normal saline to remove fragments and blood clots. Mosquito forceps gently clamped the blood clots and free fragments. A 2.0mm absorbable suture was used to cerclage around the patella’s border, clockwise and at one-third of the patellar thickness. At the same time, another absorbable suture was counterclockwise and at two-thirds of the patellar thickness from the articular surface. Pull the sutures to make sure their slippage in the soft tissue. After the initial tightening of the cerclage sutures, a double-stranded Nice knot was tied proximally to the fracture (Fig. 1).

Manipulative reduction of fractures and K-wire cerclage technique was used during the reduction. The Nice knot was gradually tightened with the gradual reduction of the bone block. During the reduction process, the surgeon's index finger directly touches the lower surface of the patella to confirm the accurate reduction of the articular surface. The reduction accuracy is continuously improved by repositioning and slight adjustment of the single bone fragments. Direct manual repositioning combined with the K-wire prying technique is a common repositioning technique. Briefly, for displaced articular surfaces and bone blocks, a Kirschner wire is inserted into the fracture end or driven into the fragment. The Kirschner wire acts as a pry, changing the position of the bone fragments and thus repositioning the fracture. The Nice knot was finally tightened after accurate fracture reduction. The patellar tendon and periosteum are reinforced with an absorbable suture. In groups A and B, cerclage titanium cable combined with a K-wire tension band was used for internal fixation, and cannulated screws were used when necessary.

### Pre- and post-operative management

Information about the patient was recorded preoperatively, including gender, age, affected side, preoperative waiting time, and fragments count based on CT 3D reconstruction. Surgical time was recorded routinely from the beginning of the skin incision to the closure of the wound. We determined the quality of the reduction by anterior-posterior and lateral radiographs the day after surgery. A step-off greater than 2 mm was considered a sign of fair reduction, and greater than 5 mm was considered poor. Anterior-posterior and lateral radiographs were obtained 1.5, 3, 6, 12 months after surgery. Fracture healing was defined as the absence of local pain or tenderness, walking well without help, and evidence of trabecular bone across the fracture line. In both groups, progressive functional exercises were started on day one postoperatively. All patients were permitted to ambulate at the 6-week follow-up fully. The function outcome was assessed by the range of motion (ROM) and the Böstman score (Excellent was defined as a score between 28 and 30; good between 20 and 27; and a score of less than 20 was considered unsatisfactory [15]. Complications were evaluated in follow-up and treated as needed.

### Statistical analyses

Continuous variables were indicated as mean ± standard deviation; categorical data were shown as a number (percentage). For a continuous variable, demographic data were assessed via the independent *t*-test for continuous data, while the *X*^2^ test was employed for the categorical data. The repeated-measures ANOVA followed by post hoc tests was carried out for the data at various follow-ups. All statistical analysis was conducted using SPSS 22.0 in this study. All statistical evaluations were two-sided. *P* < 0.05 signified statistical significance.

## Result

**Fig. 2:**
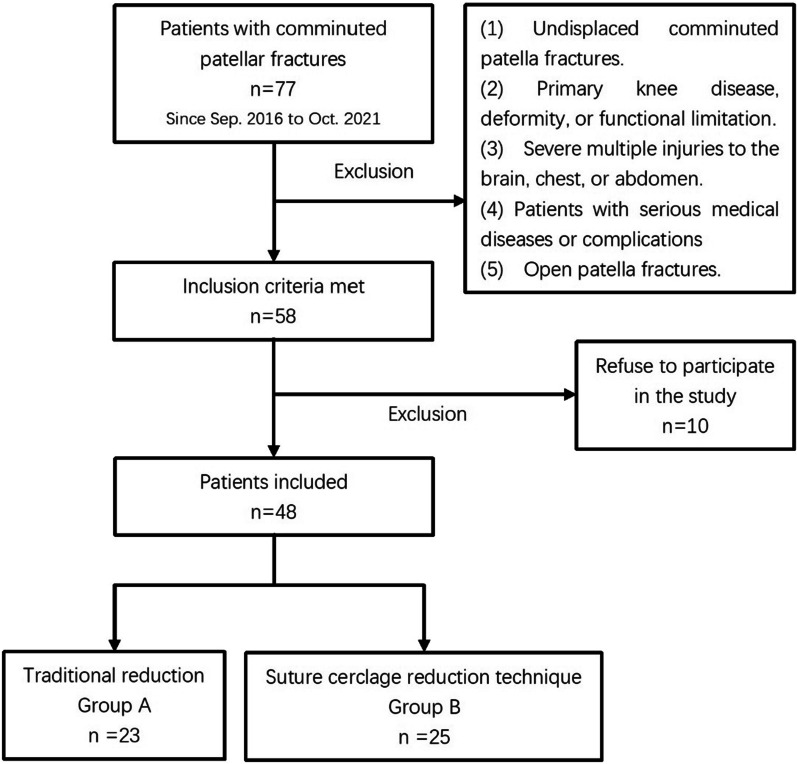
Patient enrollment

Between Sept. 2016 and Oct. 2021, 77 patella fractures with AO/OTA 34-C3 were treated at our institution. All of the study surgeons were experienced, orthopedic trauma surgeons. With the inclusion and exclusion criteria, 48 patients we included in the cohort. Patients were divided into two groups according to the method of intra-operative reduction. Twenty-three patients underwent conventional repositioning, while 25 underwent double-suture cerclage reduction technique with a Nice knot (Fig. 2).

**Fig. 3:**
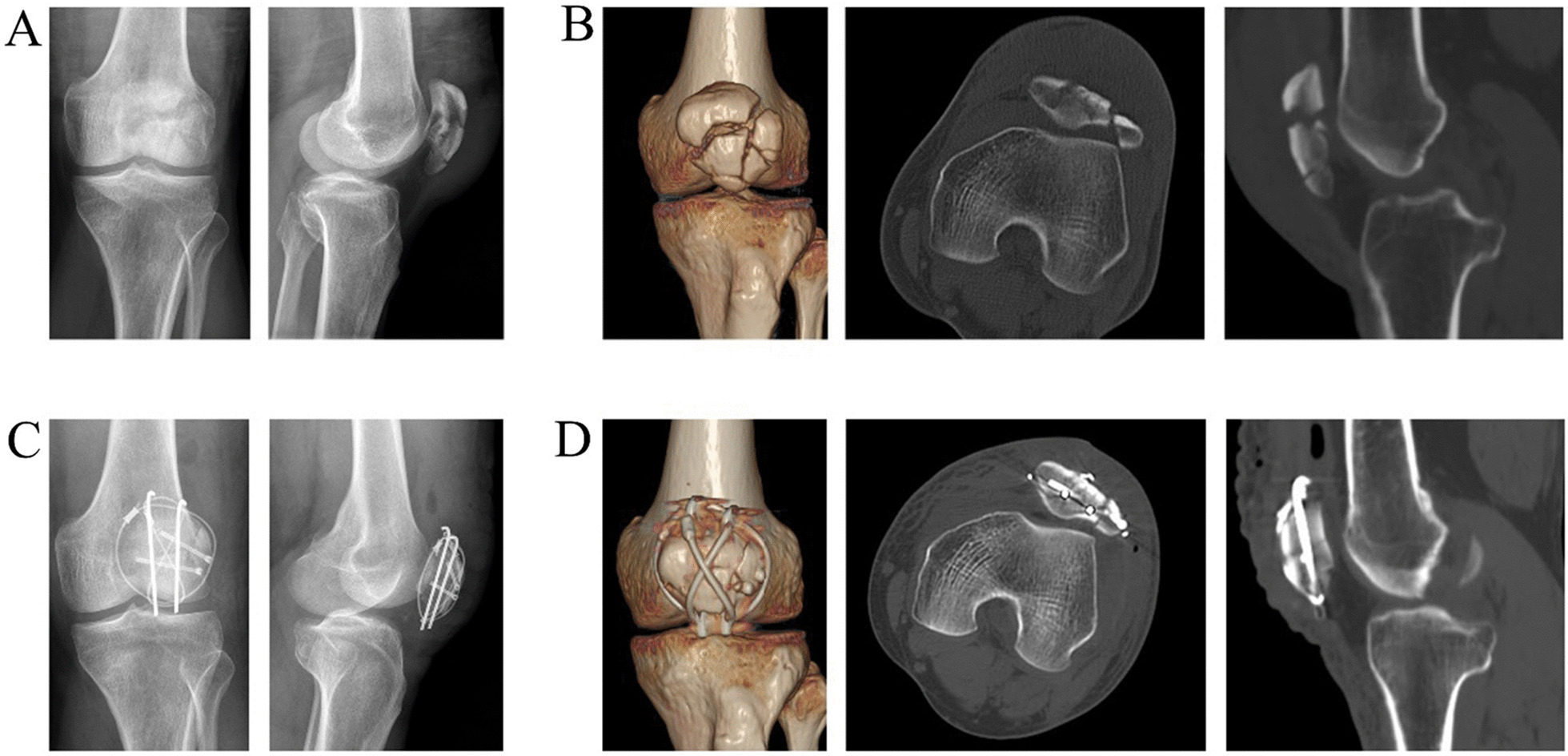
Pre- and post-operative imaging of a 57-year-old male are shown. The patient's preoperative x-ray and CT results were shown in **A**, **B**, while the post-operative x-ray and CT results were in **C**, **D**

Forty-eight patients (28 male and 20 female) were finally included in the analysis (Fig. 3). The average age of this series was 42.1 ± 11.7 (41.0 vs. 39.64). The left side was affected in 26 (14 in group A and 12 in group B), and the right was involved in 22 cases (9 in group A and 13 in group B). The number of fragments was (5.13 ± 1.18 vs. 5.32 ± 1.19). Table 1 showed no statistical difference in demographics between the two groups.

**Table 1:** Patients characteristics

	Group A	Group B	*P* value
Gender			0.807
Male	13	15	
Female	10	10	
Age	41.04 ± 13.55	39.64 ± 12.61	0.714
Affected side			0.371
Left	14	12	
Right	9	13	
Number of fragments	5.13 ± 1.18	5.32 ± 1.19	0.416

The duration of surgery, quality of reduction, ROM, functional outcome, and complications in each group are shown in Table 2. Group A had a statistically significant longer operative time than group B (89.78 ± 23.52 vs. 65.6 ± 15.38, *p* < 0.01). Statistical analysis of the quality of reduction, ROM, and knee function showed no significant differences between groups A and B. Two cases of superficial infection and one case of deep infection were identified in group A. Patients with superficial infections were cured after prolonged dressing changes. Patients with deep infections were healed after six months of post-operative surgery by removal of the internal fixation. All patients had fracture healing within six months. One patient in group B had a Kirschner wire withdrawal, and one in group A had anterior patellar soft tissue irritation. Both patients were healed after the removal of the internal fixation. Due to the small number of patients, the complications show no statistically significant differences between the two groups (*p* = 0.129). A cohort with a larger sample size is needed in future studies.

**Table 2:** Intra-operative time and patient prognosis, including Böstman score, union time, range of motion (ROM), quality of reduction, and complications

	Group A *N* = 23	Group B *N* = 25	*P* value
Duration of surgery	89.78 ± 23.52	65.6 ± 15.38	< 0.01
Böstman score	27.04 ± 2.09	27.28 ± 1.61	0.665
Union time	3.52 ± 1.08	3.48 ± 1.09	0.896
ROM	126.95 ± 8.49	128.40 ± 6.89	0.523
Quality of reduction			
Anatomical	8	9	0.797
Good	13	15	
Poor	2	1	
Complications	4	1	0.129

**Fig. 4:**
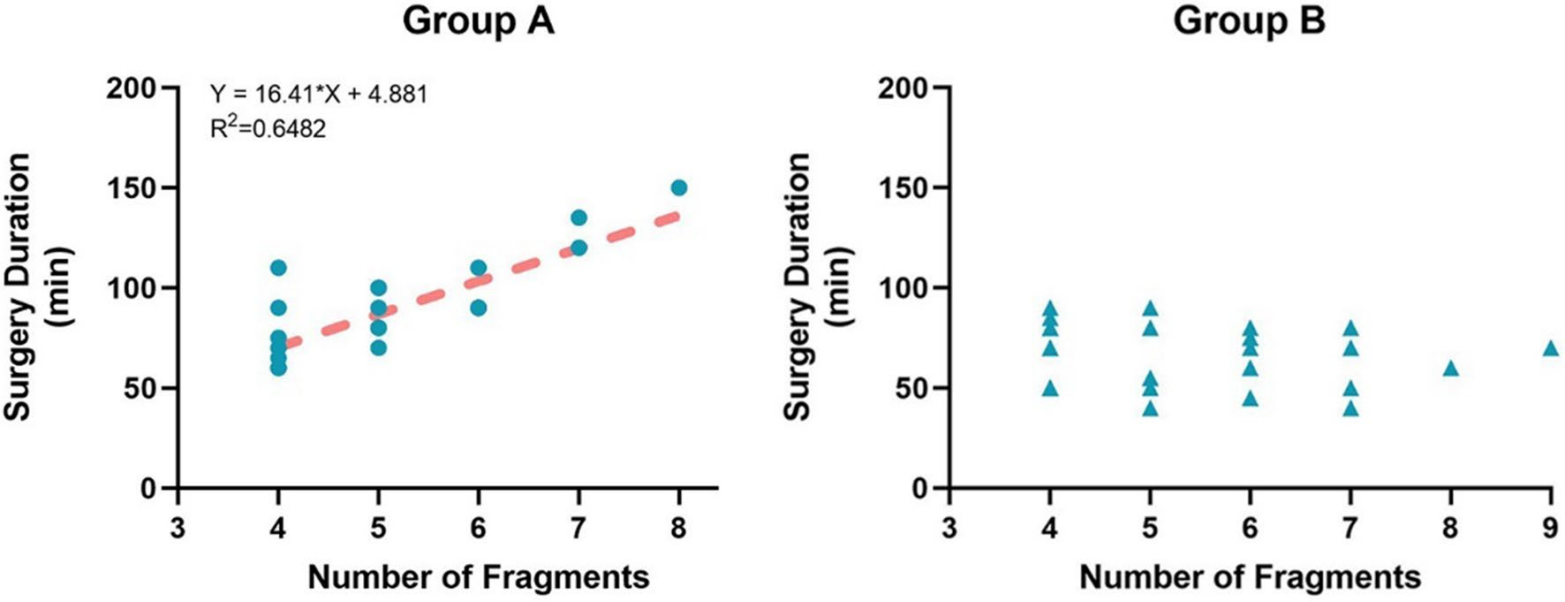
The analysis of the study on operative time and the number of fragments

The analysis of the study on operative time and the number of fragments pointed out a linear correlation in group A (*R*^2^ = 0.648). The more fracture fragments, the longer the operation time. In contrast, in group B, there was no significant correlation between operative time and the number of fracture fragments (Fig. 4).

We made a between-group comparison of operating times for different numbers of fracture blocks. It was found that when the number of fragments≥5, the operation time was significantly reduced in group B (Table 3). In comminuted patellar fractures, the suture cerclage reduction technique with Nice knots is superior to the traditional reduction technique in saving surgery time.

**Table 3:** Comparison of surgery duration in patients with different numbers of fragments between the two groups

Number of fragments	Group A	Group B	*p* value
4	73.75 ± 16.72	69.44 ± 14.99	0.607
5	85.71 ± 10.50	63 ± 18.87	0.036*
≥6	109.38 ± 22.14	63.64 ± 13.16	< 0.01*

## Discussion

The cerclage of double-stranded sutures offers a new idea of repositioning. It reduced the displacement of the fragment and offered a rough and limited fixation. The reduction accuracy is continuously improved by repositioning and slight adjustment of the single bone fragments. After the fragments were initially repositioned, the diameter of the patella became smaller. Tightening of the cerclage allows compression between the fracture fragments and further repositioning of fragments. The progressive repositioning was then performed several times. In burst comminuted patellar fractures, this technique is inverse to the mechanism of injury and often achieves an accurate reduction.

Highly comminuted patella fractures often lead to disrupted extensor mechanisms and considerable functional disability [4]. Since comminuted patella fractures are often associated with minor multi-fragmentary fractures, significant advances were found in fixation techniques, including titanium-nickel alloy, titanium cable, angle plate fixation, and low-profile mesh plate [16–19]; However, despite so many options for internal fixation, intra-operative repositioning techniques for fractures are rarely mentioned. The surgeon often spends much time and effort accurately reducing the joint surface.

Recent studies have pointed out that non-metallic fixation non-metallic or mixed implants is needed to achieve a more balanced comparison [20, 21]. As a bulky double-stranded knot, the Nice knot was proposed by Boileau and Rumian for tuberosity synthesis in proximal humerus fractures in 2010 [22]. Biomechanical properties were considered desirable with the broader use of Nice knots in recent years, making them acceptable for clinical use [12, 23, 24]. Compared to the surgical knot, the Nice knot showed a better suture strength in a biomechanical study [10, 25]. Intra-operative temporary fixation by suture with a Nice knot was reported to reduce fragments, shorten the operation time, and effectively treat transverse patellar fractures [26]. In this study, the Nice knot combined with double-suture cerclage provides sufficient tightening strength during fracture repositioning. Intermittent tightening of the sutures compresses the fracture fragments.

Mostly, a longer surgery duration means a more complicated fracture, a higher technical demand, a more extensive soft tissue stripping, and a longer exposure time of surgical wounds to airborne dust [27]. Although our study did not demonstrate the statistical significance of the technique in reducing clinical complications, a shorter surgery duration could reduce the incidence of infection, which needs to be confirmed by reports from a more extensive study.

Several limitations of the current study should be recognized. First, the sample size was small. The sample size for this experiment was small. A larger sample size would help to come to a statistically significant conclusion about clinical complications. In our future work, we will continue to recruit patients and increase the sample size to confirm this result. Second, the follow-up period is slightly shorter, with consequences for traumatic arthritis and long-term follow-up taking longer to detect. Third, this is a retrospective study. The intra-operative surgeons’ choice of repositioning method inevitably biases the results. An RCT study could avoid this bias and increase confidence in the experimental results.

## Conclusion

In this study, double-suture cerclage reduction technique with Nice knot shows great advantages for displaced highly comminuted patella fractures. This technique simplifies the operation and saves surgical time, which is helpful for clinical practice.

## Data Availability

The datasets used and analyzed during the current study are available from the corresponding author on reasonable request.
